# Supplementary material for: The neglected role of relative humidity in the interannual variability of urban malaria in Indian cities
Source: Nat Commun. 2022 Jan 27;13:533. doi: 10.1038/s41467-022-28145-7 (PMC8795427; doi:10.1038/s41467-022-28145-7)
Supplement: Supplementary file 1 — Supplementary Information [file 41467_2022_28145_MOESM1_ESM.pdf]

Supplementary information

The neglected role of relative humidity in the interannual variability of urban malaria in Indian cities

Santos-Vega, M, Martinez, P.P, Vaishnav, K.G., Kohli, V., Desai, V., Bouma, M.J., Pascual, M.

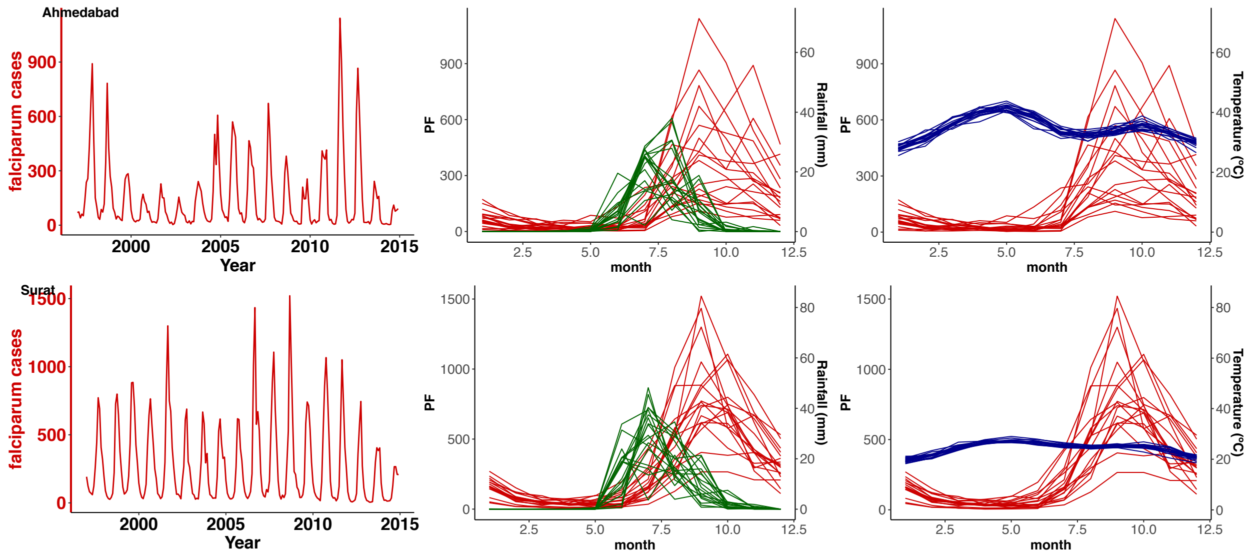

Suppl. Figure 1. Monthly case data for *P. falciparum* (in red) from 1997 to 2014 are shown for Ahmedabad (top) and Surat (bottom) together with the corresponding time series of relative temperature and rainfall (blue and green, respectively). The corresponding seasonality of these variables is illustrated in the right panels by superimposing the time series for different years as a function of month.

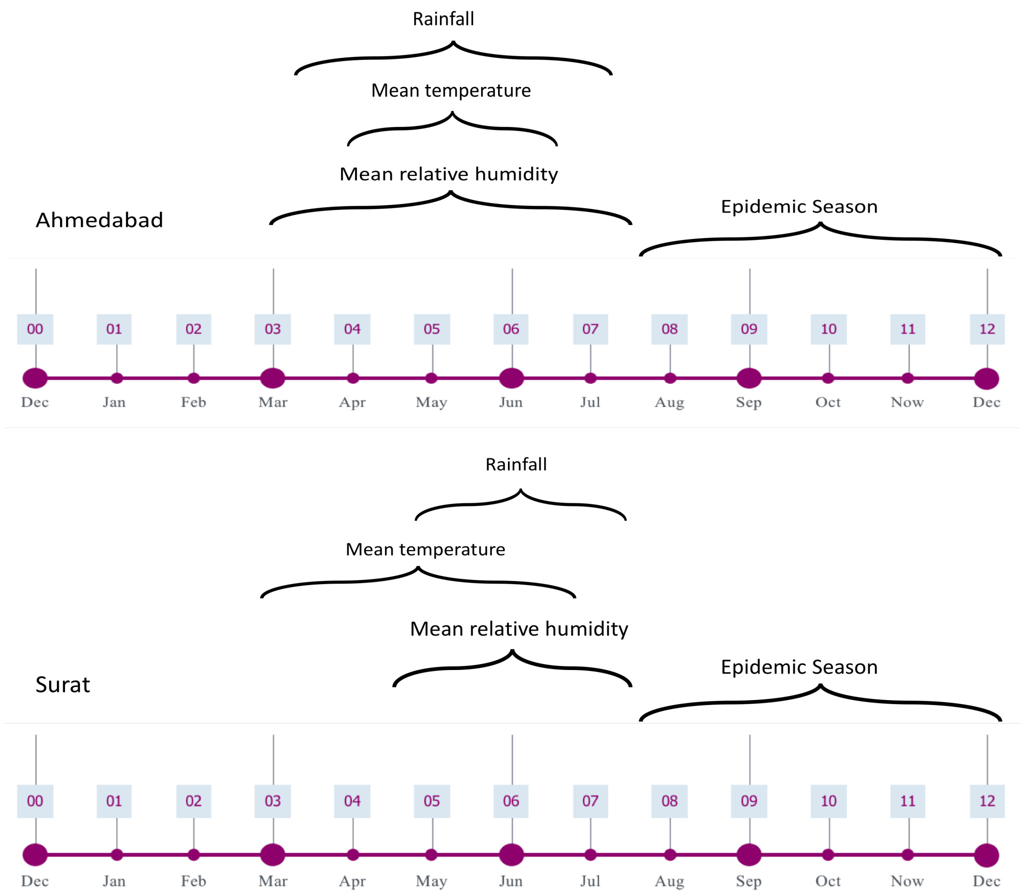

Suppl. Figure 2. Diagram showing the main malaria season (August to December) and the selected windows of time for each of the climate covariates, for both cities Ahmedabad (top) and Surat (bottom). The critical windows for the covariates were selected on the basis of the highest correlations with the aggregated cases during the transmission season, and the condition that the time interval precedes that season. These windows were obtained for: a) average relative humidity for the four and three months preceding the epidemic season for each city, respectively, b) average temperature from April to July for Ahmedabad and March to June for Surat and c) cumulative rainfall from March to July for Ahmedabad, and May to July for Surat.

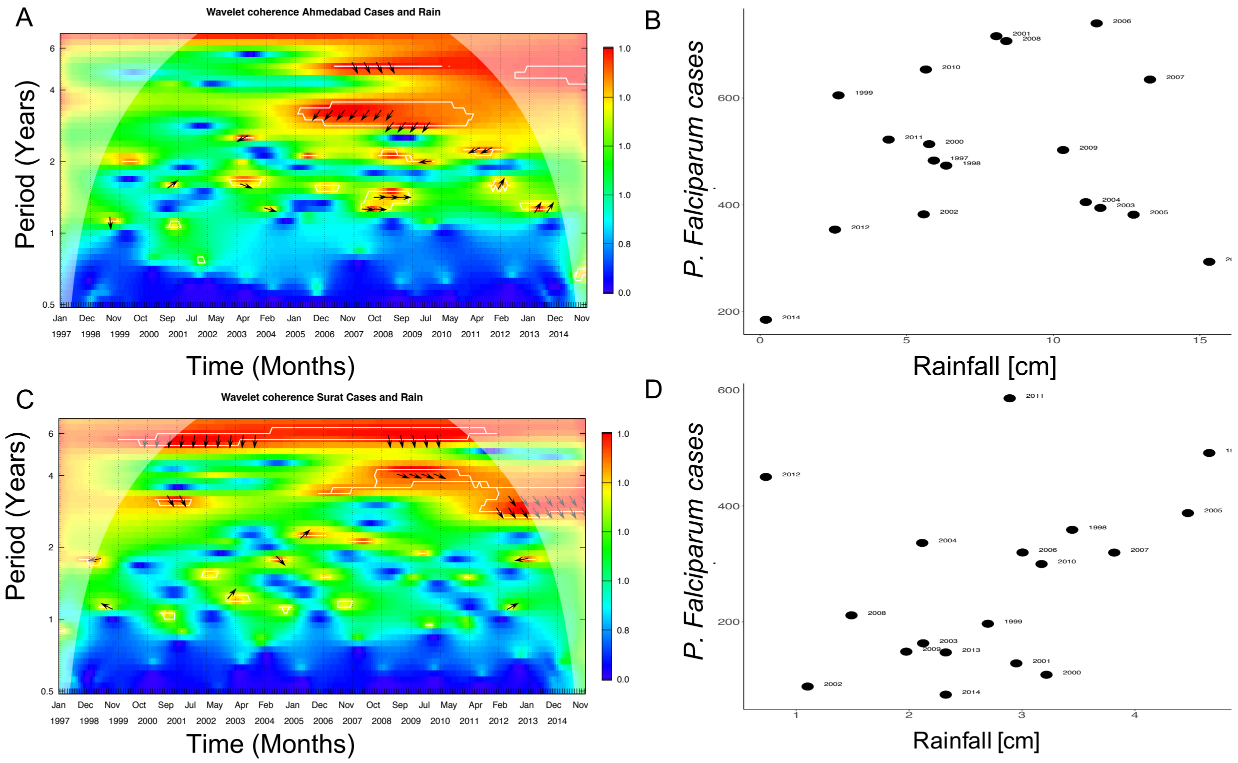

Suppl. Figure 3. Patterns of association between malaria cases and rainfall for Ahmedabad (top) and Surat (bottom). (A) and (C) The color panels consider associations in the frequency domain with cross-coherence wavelet spectra. Cross-coherence varies between 0 and 1 in a color scale from blue to red with the lines indicating 5% significance levels. Only regions within these lines exhibit significant cross-coherence at those levels. The shaded region corresponds to periods and times that are affected by the boundaries and are outside the so-called cone of significance**.** (The climate variables have been filtered for these analyses to remove seasonality and focus on the association with malaria at interannual time scales). (B and D) Scatter plots demonstrate the weaker correlations between malaria cases and the rainfall compared to humidity shown in Figure 1 in the main text. The Pearson correlation values are R=0.42 for Ahmedabad and R=0.23 for Surat. The total cases during the transmission season from August to November are shown as a function of the average rainfall during a critical window preceding this season. These windows are indicated in Figure S2.

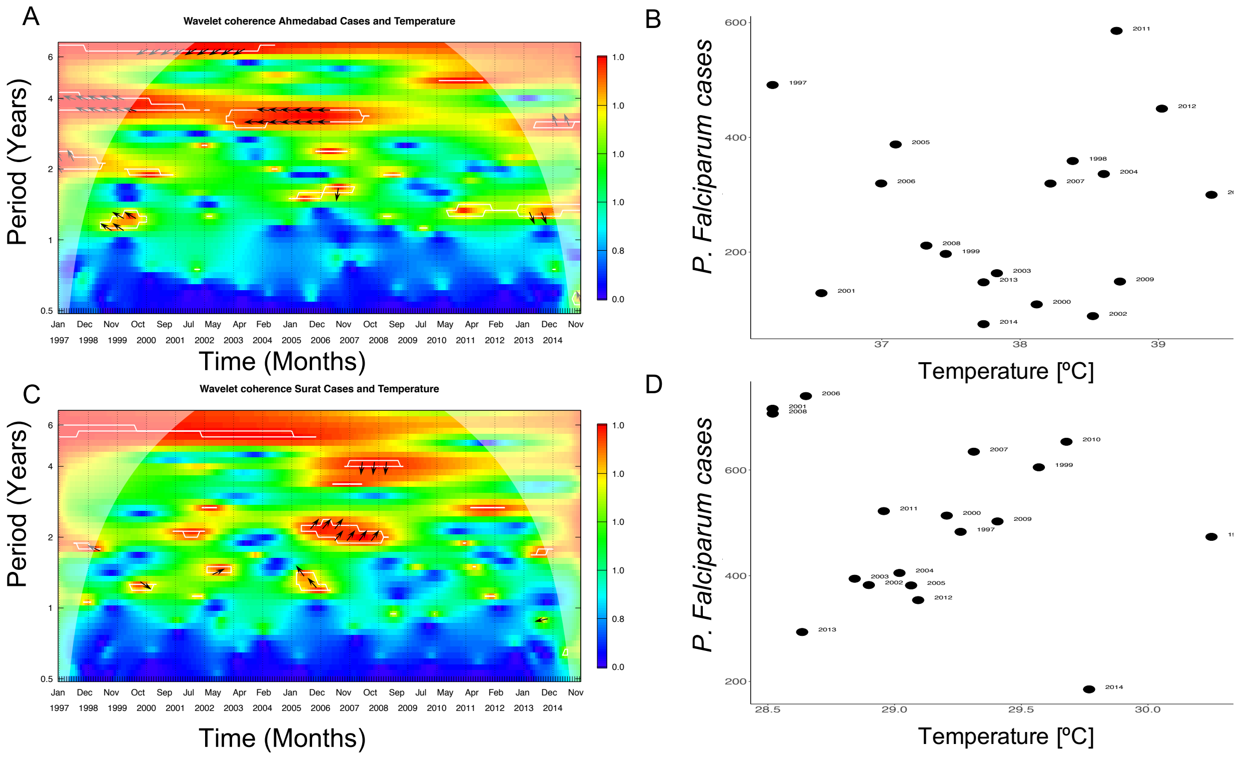

Suppl. Figure 4. Patterns of association between malaria cases and temperature for Ahmedabad (top) and Surat (bottom). (A) and (C). The color panels consider associations in the frequency domain with cross-coherence wavelet spectra. Cross-coherence varies between 0 and 1 in a color scale from blue to red with the lines indicating 5 and 10% significance levels. Only regions within these lines exhibit significant cross-coherence at those levels. The shaded region corresponds to periods and times that are affected by the boundaries and are outside the cone of significance**.** (The climate variables have been filtered to remove seasonality and focus on interannual variability). (B) and (D) Scatter plots demonstrate the weaker correlations between malaria cases and the temperature compared to humidity shown in Figure 1 in the main text. The Pearson correlation values are R=0.26 for Ahmedabad and R=0.39 for Ahmedabad. The total cases during the transmission season from August to November are shown as a function of the average temperature in a critical window preceding this season and shown in Figure S2.

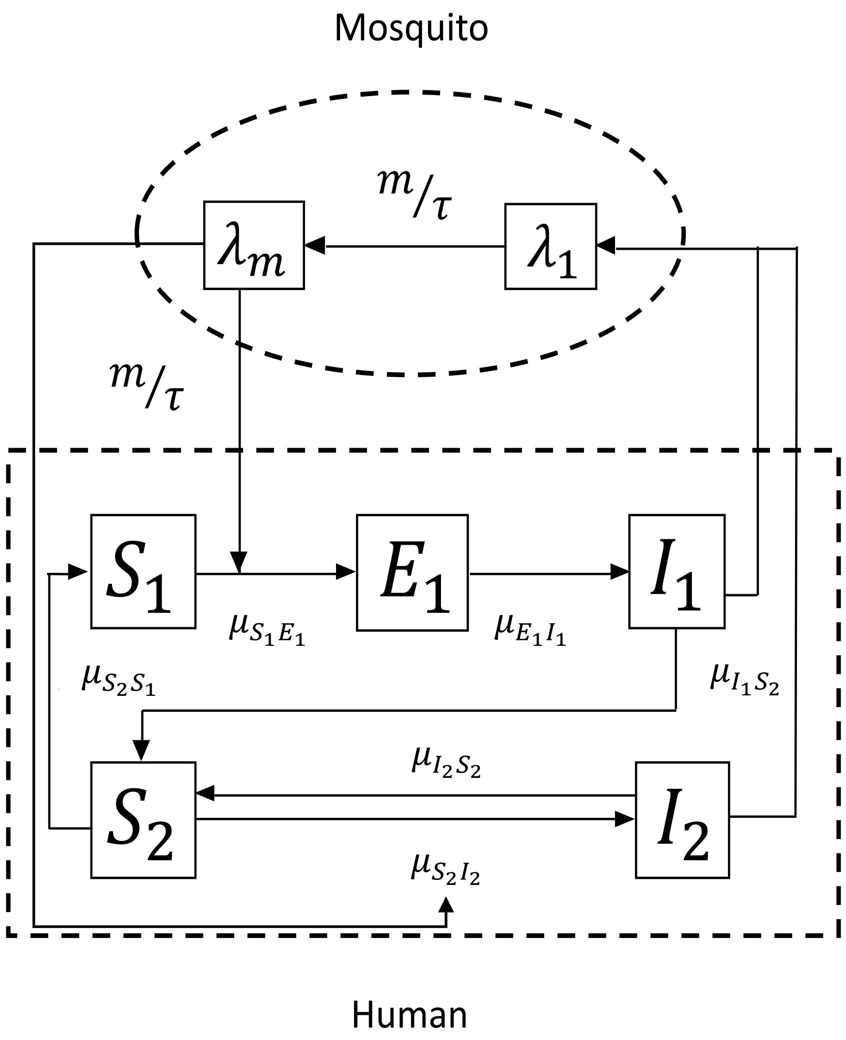

Suppl. Figure 5. Model diagram. Flow diagram of the SDE model which subdivides the total population human population into S1 (susceptible), E (exposed, carrying a latent infection), I1 (symptomatic infected and infectious), I2 (asymptomatic infected and infectious), and S2 (recovered sub patent, i.e., having some resistance to reinfection). The chain of classes implicitly accounts for the mosquito component by implementing a distributed delay in the force of infection experienced by a susceptible host, as explained in the Methods. Arrows indicate transitions between classes X and Y with the corresponding rate m. The model is formalized in Eqs. 1–6.

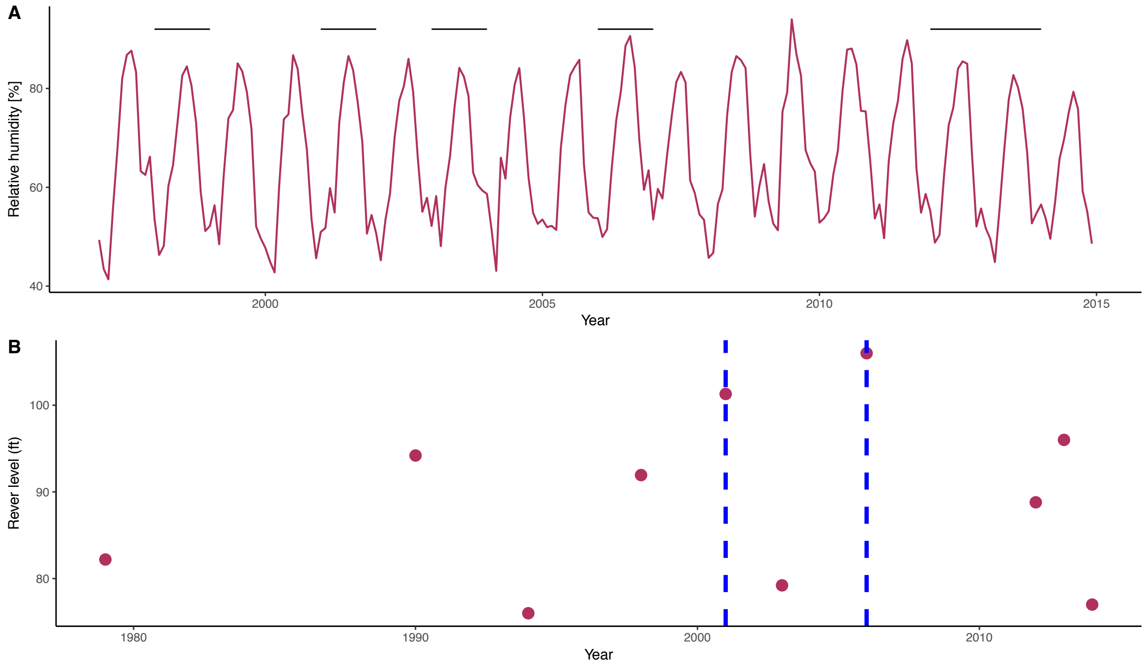

Suppl. Figure 6. Panel A shows monthly relative humidity for the city of Surat from 1997 to 2014 with flooding years indicated by black segments. Panel B shows levels of the Tapi river in Surat for flooding years. The years of 2001 and 2006 are highlighted with broken blue lines as they correspond to the timing of the major floods, with river levels above 100 ft. Note that the time axes for the two plots have different scales and the time series for river levels starts earlier than that for relative humidity.

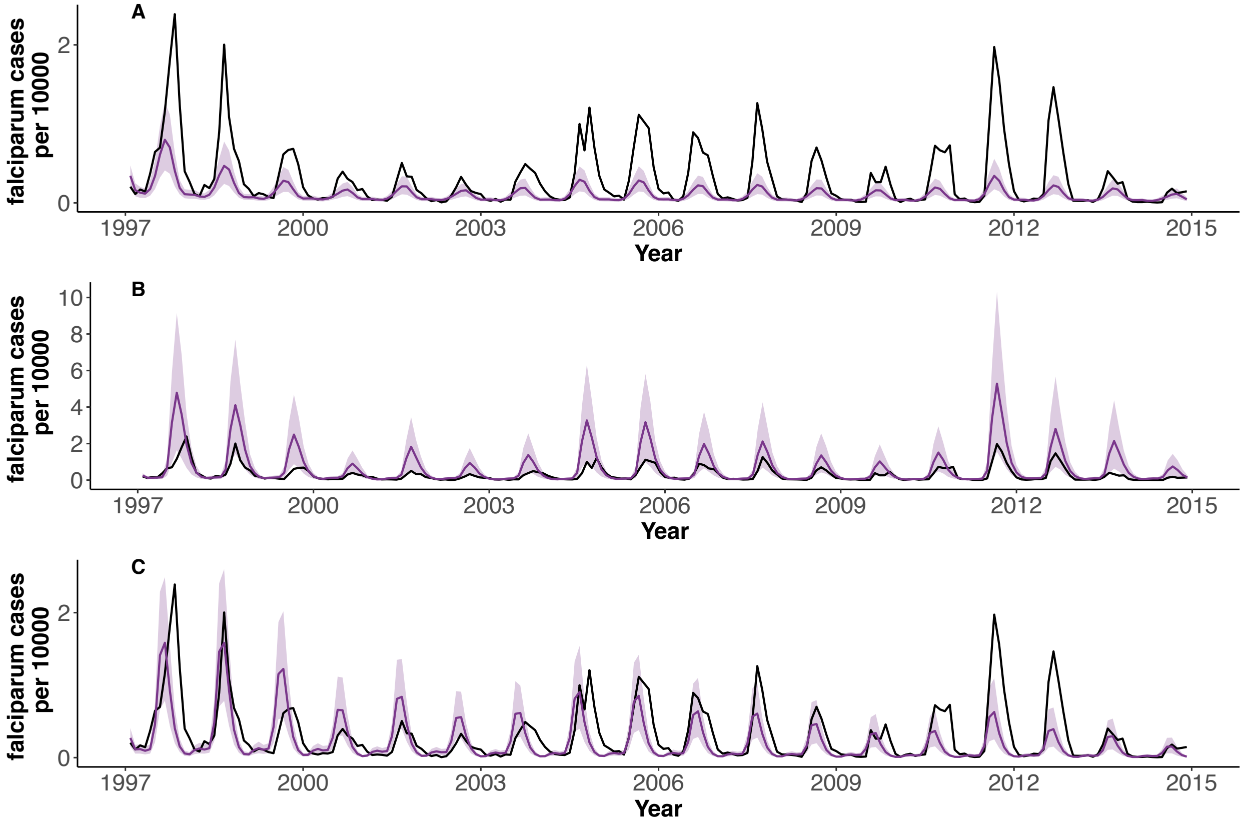

Suppl. Figure 7. Comparison of observed and simulated monthly cases with the alternative models for Ahmedabad. The panels show simulation results for the model with no climate covariate (A), and those driven by temperature and rainfall (B and C) respectively. The time series for the observed cases are in black and the mean of 1,000 model simulations is indicated in purple. The intervals between the 10 % and 90 % percentiles of the simulated trajectories are shaded in light purple. The simulated cases are not next step predictions but the predicted values from forward simulations of the model for the whole 20 years' study period starting with estimated initial conditions.

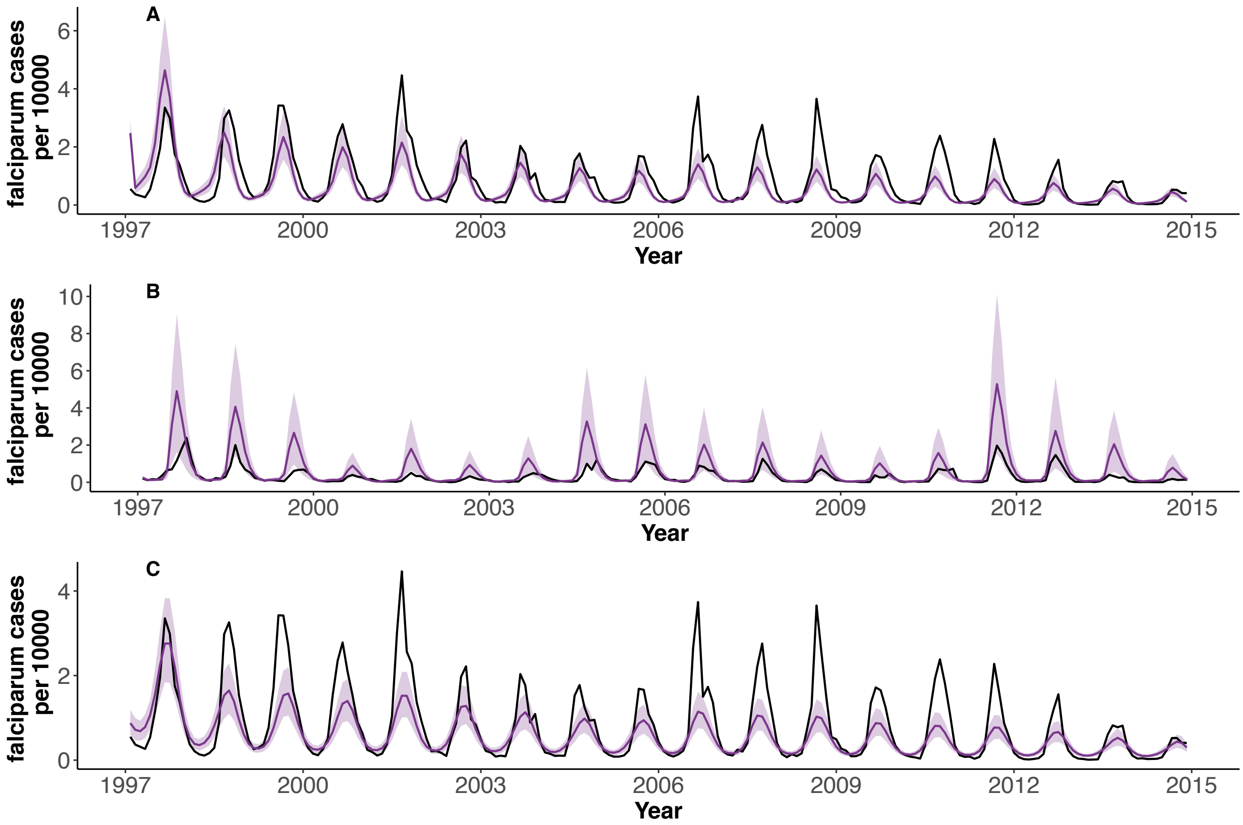

Suppl. Figure 8. Comparison of observed and simulated monthly cases with the alternative models for Surat. The panels show simulation results for the city of Surat, from top to bottom respectively for the model with no climate covariate (A), the one driven by temperature (B) and the one l driven by rainfall (C). The time series for the observed cases are in black and the mean of 1,000 model simulations is given in purple. The intervals between the 10 % and 90 % percentiles of the simulated trajectories are shaded in light purple. The simulated cases are not next step predictions but the predicted values from forward simulations of the model for the whole 20 years' study period starting with estimated initial conditions.

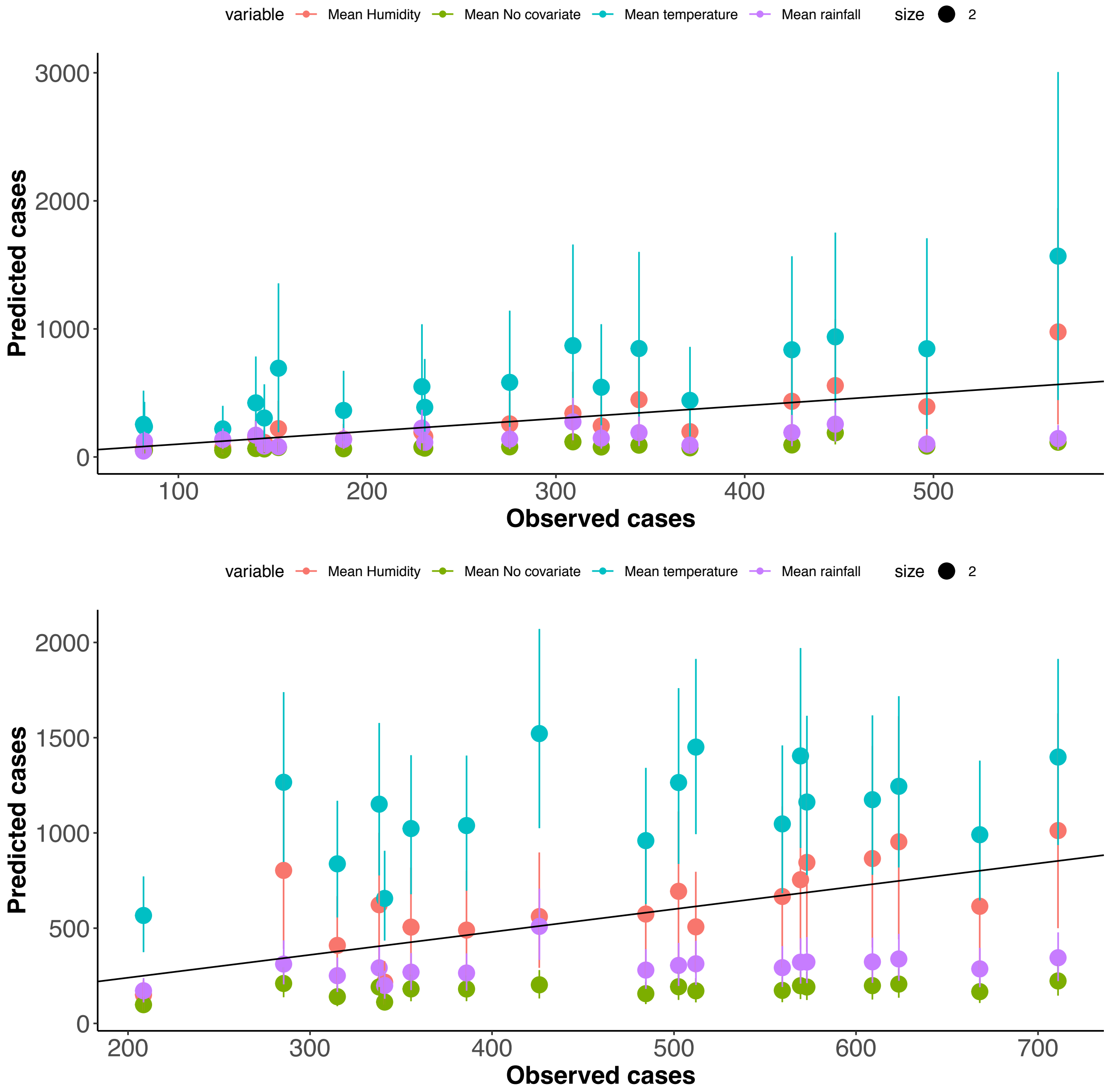

Suppl. Figure 9. Scatter plots for the sum of the mean predicted cases against the sum of the observed cases during the epidemic season of a given year, for Ahmedabad (Top) and Surat (Bottom). In the plots, red dots represent the results from the humidity model, purple dots those for the rainfall-driven model, blue dots those for the temperature -driven model, and green dots those for the model with no covariate The line represents the identity line indicating the expectation when predictions equal observations.

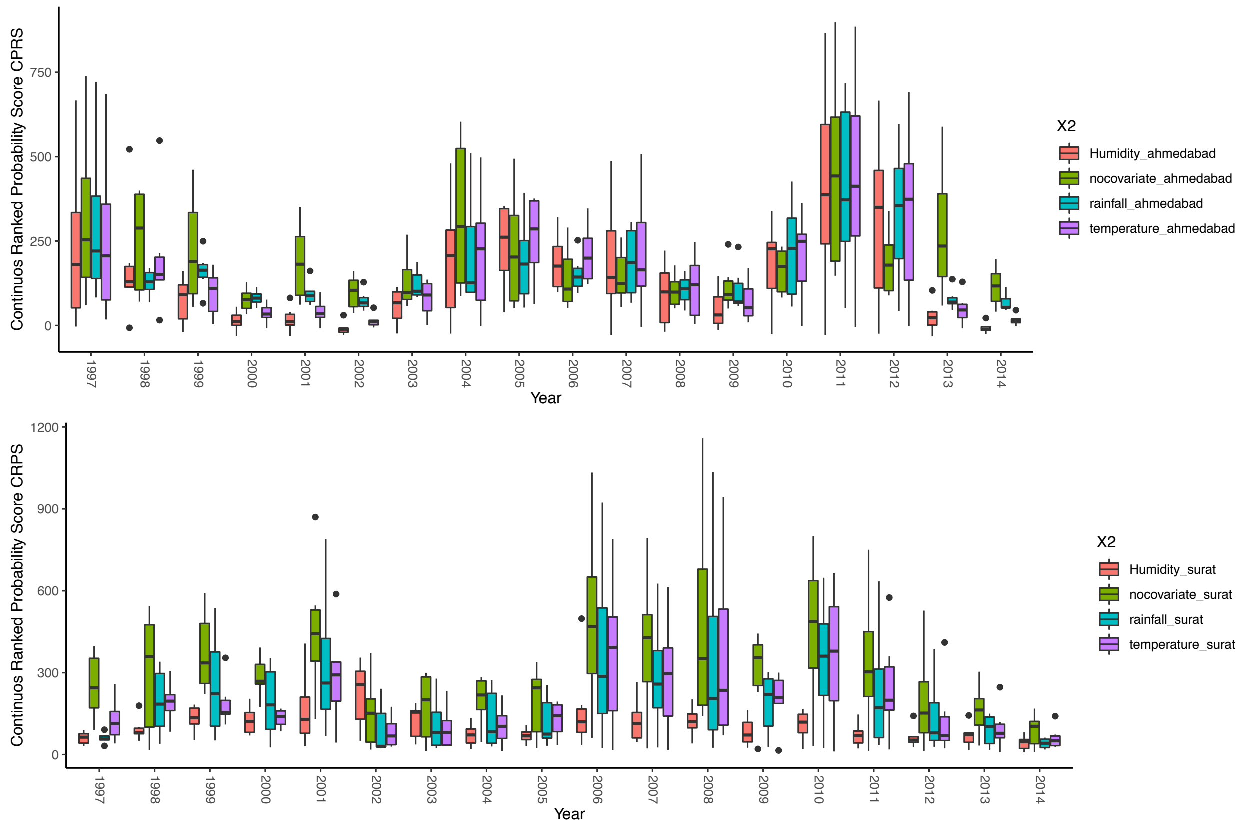

Suppl. Figure 10. This figure shows the continuous ranked probability score (CRPS) of the four models evaluated each year for the prediction based on n=1000 model realizations. The CRPS compares the empirical distribution of a prediction to a scalar observation. Smaller scores indicate better skill. (The boxplots display the minimum, 25th percentile, median, 75th percentile and maximum, as is standard).

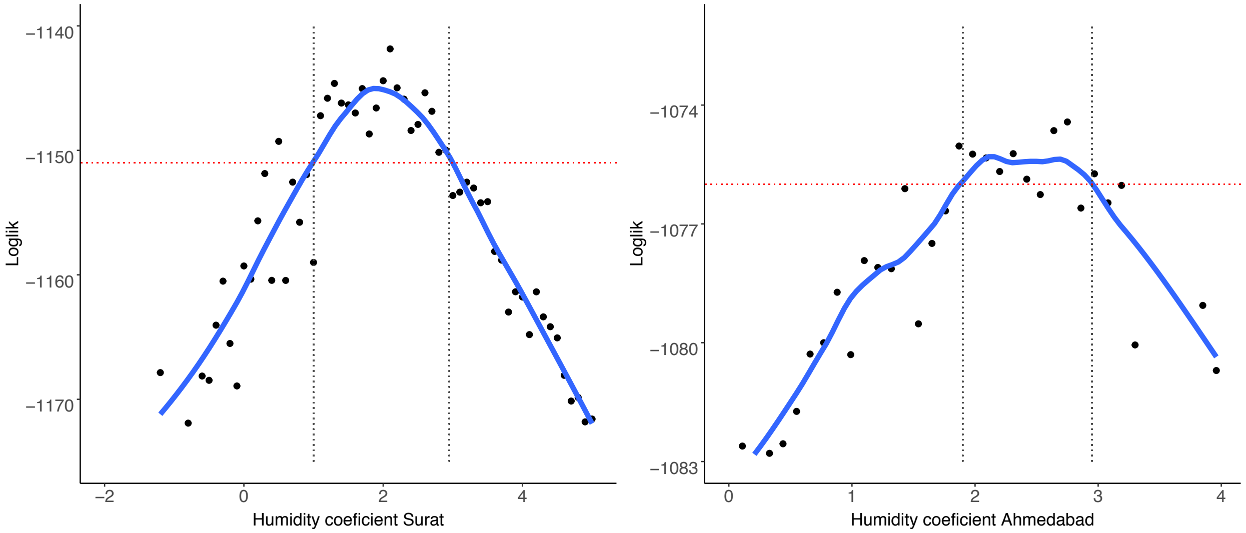

Suppl. Figure 11. Coefficient corresponding to the interannual effect of relative humidity on the transmission rate for Surat (left) and Ahmedabad (right). Likelihood profile curves are shown, with the intersections between the likelihood curve (solid blue line) and the horizontal red dotted line, two likelihood units below the MLE, providing the 95% CI of this parameter for each region.

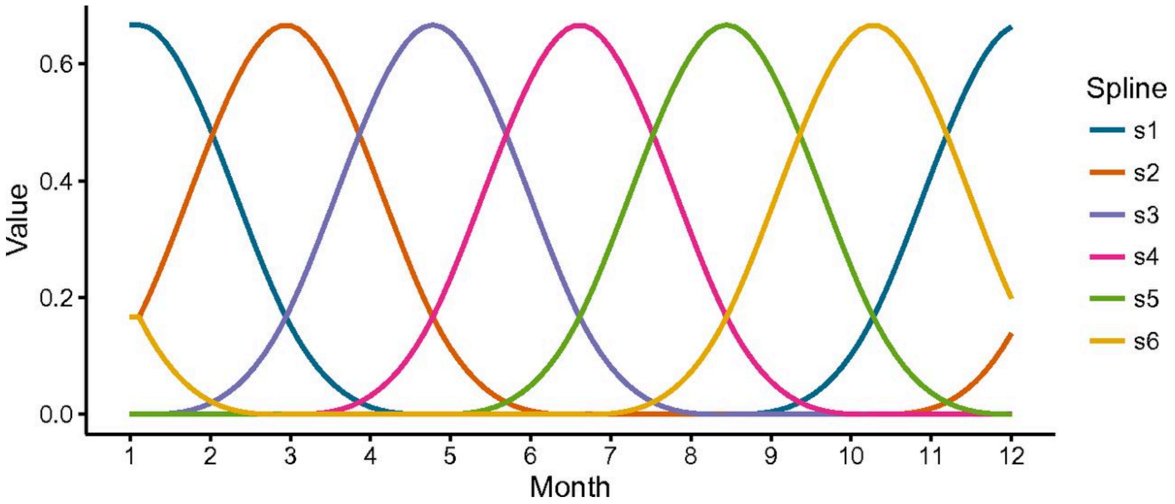

Suppl. Figure 12. Periodic splines. The six beta splines (s1…s6) used to capture seasonality in the expression of the transmission rate.

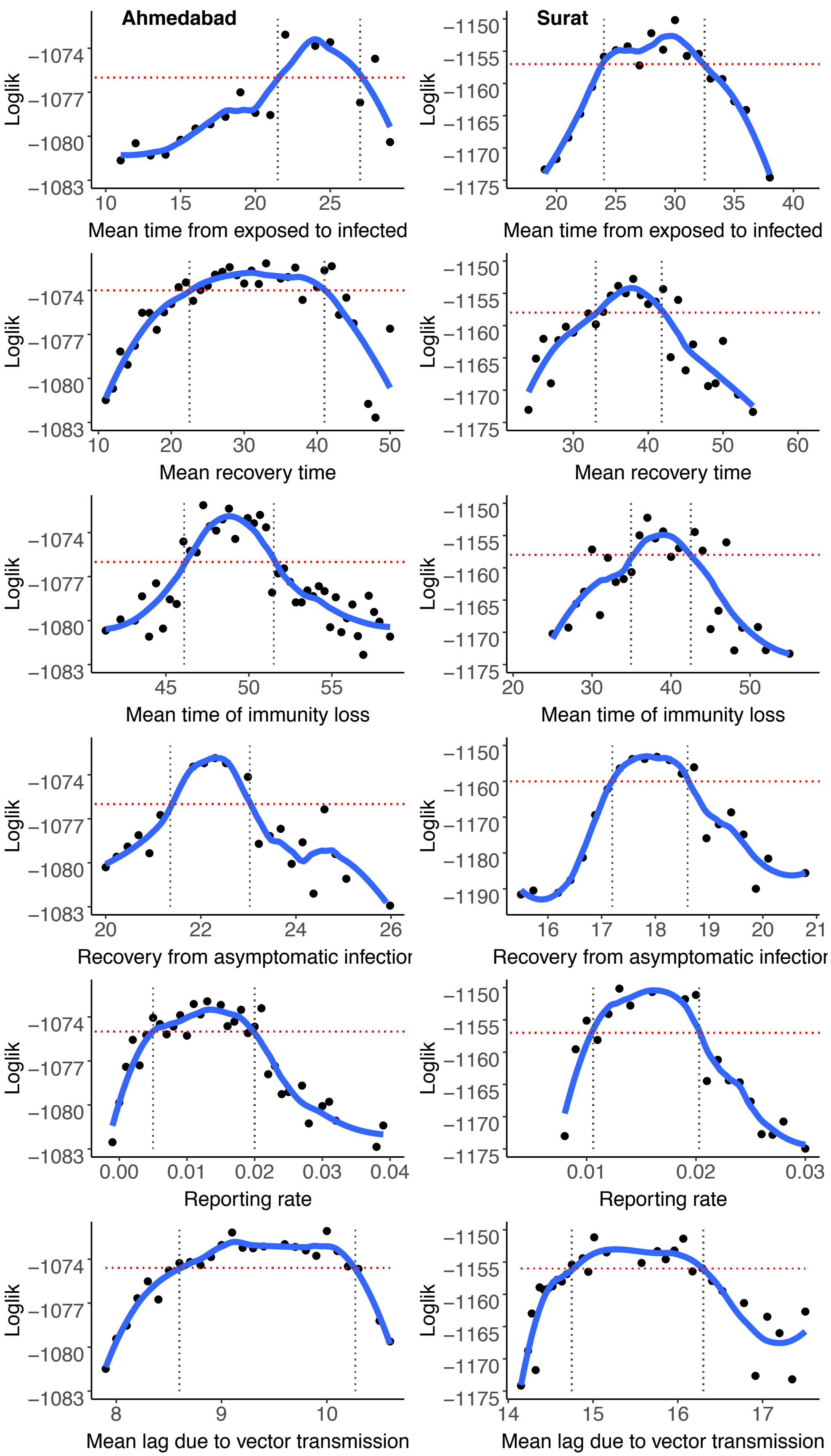
Ahmedabad
Surat
Loglik
Mean time from exposed to infected
Mean recovery time
Mean time of immunity loss
Recovery from asymptomatic infectior
Reporting rate
Mean lag due to vector transmission

Suppl. Figure 13. Log-likelihood profiles for epidemiological parameters. The intersections between the red dashed line and the vertical dashed lines provide the 95% CI for each parameter.

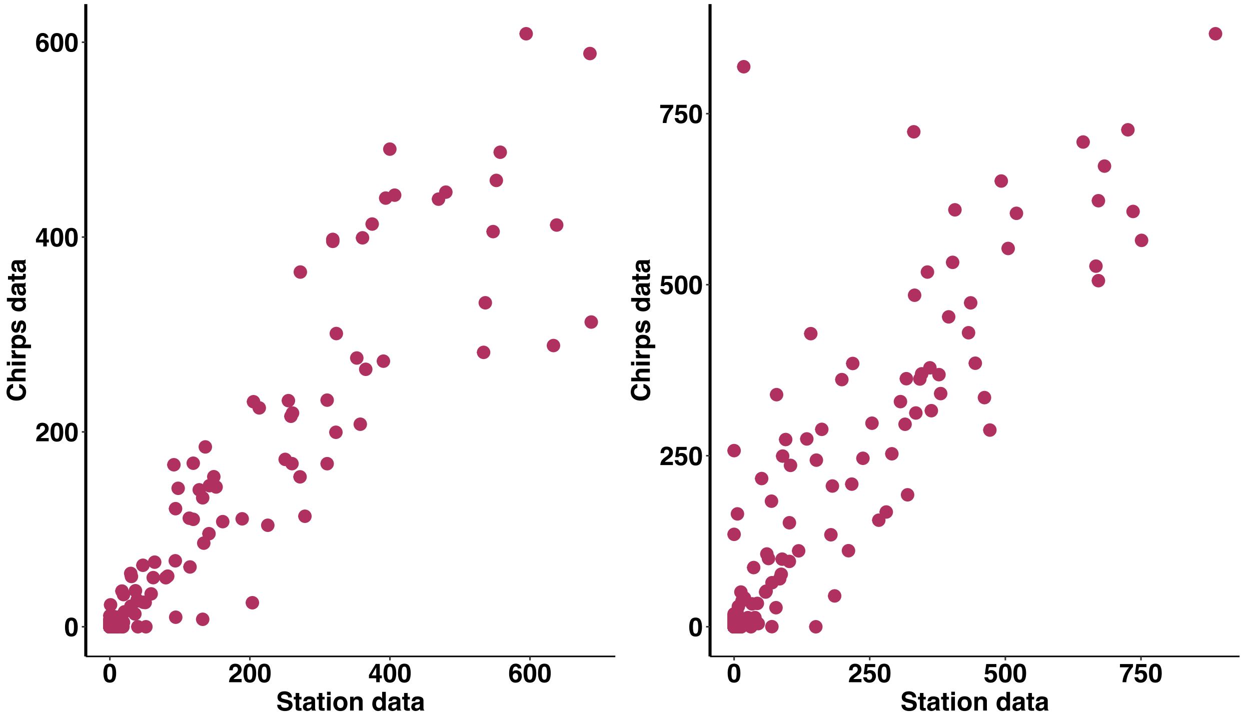

Suppl. Figure 14. Comparison between the gridded climate product and the meteorological station data.

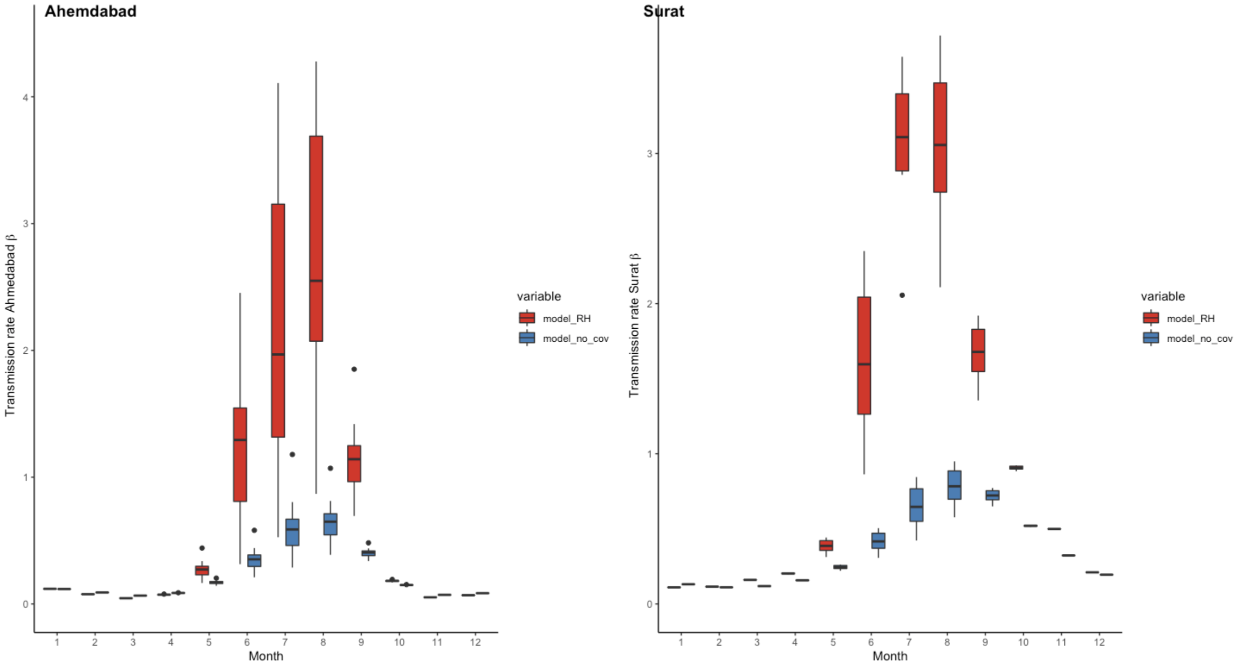

Suppl. Figure 15. Comparisons of the transmission rates estimated for the model with no covariates (with only the splines used in the model) and for the best model with a climate covariate (relative humidity) (see expression of the transmission rate in Methods). The boxplots are computed on the basis of 1000 simulations (and display the minimum, 25th percentile, median, 75th percentile and maximum).

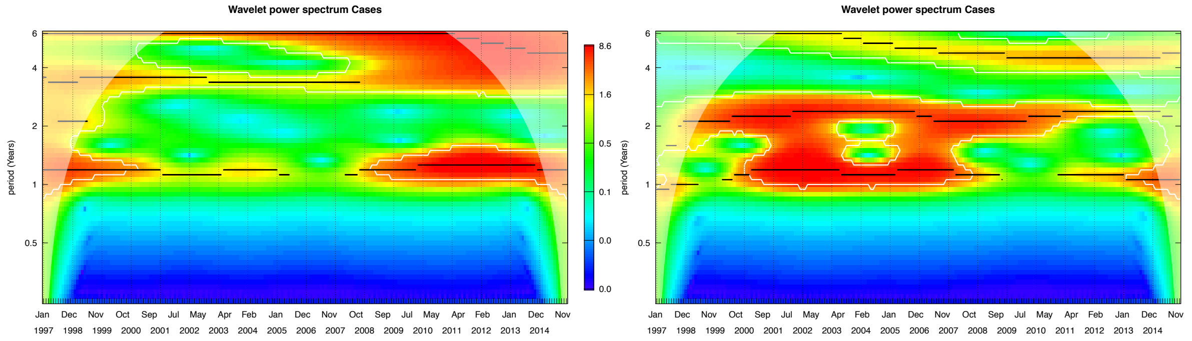

Suppl. Figure 16. Wavelet analysis of the monthly number of reported cases of malaria cases in Ahmedabad (left) and Surat (right).

parameter						
Surat	-3.491[- 5.161- - 1.476]	-1.8893 [- 0.7245- - 0.4032]	0.7715 [- 1.5823- 2.0431]	0.9888 [0.4099- 1.4646]	-0.4090 [- 2.2127 - 0.9694]	-3.874 [- 5.703 - 1.540]
Ahmedabad	-2.5062 [- 4.0813 -- 0.7151]	- 1.4095[- 3.4383- 0.6278]	1.9280 [0.9239- 4.5710]	0.2192[- 1.7202- 2.1962]	- 0.5682[- 2.8054-- 0.9221]	- 3.317 [-5.508- -1.200]

Suppl. Table 1. Parameter estimates for the coefficients of each of the six splines defining the seasonality of the transmission rate

Covariate	Ahmedabad	Surat
Humidity	0.72	0.69
Rainfall	0.42	0.23
Temperature	0.26	0.39

Suppl. Table 2. Pearson correlation of the best lagged correlations identified between the climate covariates and the malaria epidemic peak.

Description	**Unit**	**parameter**	**Ahmedabad**	**CI**	**Surat**	**CI**
Mean time from exposed to infected	Days	$1/\mu_{EI_1}$	25.1	[19.8-25.9]	27.4	[25-32]
Mean recovery time	Days	$1/\mu_{EI_1}$	29.92	[28.2-35.5]	36.5	[32-43]
Mean time of immunity loss	Days	$1/\mu_{I_1S_2}$	47.05	[46.57-51.12]	35.24	[34.72-38.11]
Recovery from asymptomatic infection	Days	$1/\mu_{I_2S_2}$	21.94	[20.11-25.31]	17.95	[15.72-21.946]
Case reporting fraction	Days	ρ	0.016	[0.10-0.018]	0.017	[0.012-0.019]

Suppl. Table 3. Parameter estimates and confidence intervals for both cities for the model fitted to the shorter period up to 2009. (The average human lifespan was fixed at 50 years). The MLE values are close to those obtained for the whole time series, up to 2014. Compare values to those of Table 2 in the main text.
