# Supplementary material for: The neglected role of relative humidity in the interannual variability of urban malaria in Indian cities
Source: Nat Commun. 2022 Jan 27;13:533. doi: 10.1038/s41467-022-28145-7 (PMC8795427; doi:10.1038/s41467-022-28145-7)
Supplement: Supplementary file 3 — Reporting Summary [file 41467_2022_28145_MOESM3_ESM.pdf]

nature portfolio

Corresponding author(s): Mercedes Pascual

Last updated by author(s): November 30, 2021

Reporting Summary

Nature Portfolio wishes to improve the reproducibility of the work that we publish. This form provides structure for consistency and transparency in reporting. For further information on Nature Portfolio policies, see our Editorial Policies and the Editorial Policy Checklist.

Statistics

For all statistical analyses, confirm that the following items are present in the figure legend, table legend, main text, or Methods section.

n/a	Confirmed	
☐	☒	The exact sample size (*n*) for each experimental group/condition, given as a discrete number and unit of measurement
☐	☒	A statement on whether measurements were taken from distinct samples or whether the same sample was measured repeatedly
☐	☒	The statistical test(s) used AND whether they are one- or two-sided<br>*Only common tests should be described solely by name; describe more complex techniques in the Methods section.*
☐	☒	A description of all covariates tested
☒	☐	A description of any assumptions or corrections, such as tests of normality and adjustment for multiple comparisons
☐	☒	A full description of the statistical parameters including central tendency (e.g. means) or other basic estimates (e.g. regression coefficient) AND variation (e.g. standard deviation) or associated estimates of uncertainty (e.g. confidence intervals)
☐	☒	For null hypothesis testing, the test statistic (e.g. *F*, *t*, *r*) with confidence intervals, effect sizes, degrees of freedom and *P* value noted<br>*Give P values as exact values whenever suitable.*
☒	☐	For Bayesian analysis, information on the choice of priors and Markov chain Monte Carlo settings
☒	☐	For hierarchical and complex designs, identification of the appropriate level for tests and full reporting of outcomes
☒	☐	Estimates of effect sizes (e.g. Cohen's *d*, Pearson's *r*), indicating how they were calculated

Our web collection on statistics for biologists contains articles on many of the points above.

Software and code

Policy information about availability of computer code

Data collection	NA
Data analysis	The model fitting (parameter inference) and simulations were implemented using the R-package pomp version 3.3.0.0 with R version 4.0.4 (2021-02-15). The wavelet analyses were implemented with the R-package WaveletComp 1.1.

For manuscripts utilizing custom algorithms or software that are central to the research but not yet described in published literature, software must be made available to editors and reviewers. We strongly encourage code deposition in a community repository (e.g. GitHub). See the Nature Portfolio guidelines for submitting code & software for further information.

Data

Policy information about availability of data

All manuscripts must include a data availability statement. This statement should provide the following information, where applicable:

- Accession codes, unique identifiers, or web links for publicly available datasets
- A description of any restrictions on data availability
- For clinical datasets or third party data, please ensure that the statement adheres to our policy

The monthly reported malaria cases and values of the different climate covariates can be found in the data file accompanying the code at https://github.com/pascualgroup/Humidity_malaria (doi: 10.528/zenodo.5734922)

Field-specific reporting

Please select the one below that is the best fit for your research. If you are not sure, read the appropriate sections before making your selection.

☐ Life sciences ☐ Behavioural & social sciences ☒ Ecological, evolutionary & environmental sciences

For a reference copy of the document with all sections, see nature.com/documents/nr-reporting-summary-flat.pdf

Ecological, evolutionary & environmental sciences study design

All studies must disclose on these points even when the disclosure is negative.

Study description	This study relies on process-based models for the population dynamics of malaria driven by different climate factors and on their fitting to time series data of reported malaria cases in two cities of India, to address the importance of relative humidity to the interannual variability of seasonal epidemics. The different models are respectively driven by relative humidity, rainfall and temperature; a baseline that only includes seasonality in the transmission rate is also considered. Formal comparison of the models based on likelihood indicate that the model including relative humidity best explains the data. This model also exhibits the highest predictive ability.
Research sample	The data used in this study originates from the malaria surveillance conducted in the two cities, namely Ahmedabad and Surat (in India), by the respective Municipal Corporations. The data consists of two time series of monthly reported cases collected from 1997 to 2014. Thus each time series consists of 216 months. This is the relevant length to consider the interannual variation in the seasonal epidemics. We have 18 seasons in each time series, and the characteristic time scales of interannual variation (about 2 and 4 years) are sufficiently represented given the length of these time series.
Sampling strategy	NA. We did not sample the data. We used existing time series.
Data collection	The malaria data were not collected by us; monthly malaria cases were collected from 1997 to 2014 by the respective Municipal Corporations of the cities of Ahmedabad and Surat. These epidemiological data result from two kinds of surveillance: (a) the collection of blood slides from fever patients by house-to-house visits by a health worker and examination of these slides for positive malaria parasites at the Primary/Community Health Center (active surveillance); (b) examination of blood slides from fever patients reporting directly to the Primary/Community Health Center (passive surveillance). Both types of data are pooled into a temporal record for each city. We used climate data of monthly relative humidity, rainfall and temperature for the same 18 years, obtained from local weather stations recorded at a local weather station within each city, supplied by the Indian Meteorological Department in Pune (India) and verified in the GHCN network of climate data (https://www.ncdc.noaa.gov/ghcn-daily-description).
Timing and spatial scale	As described above, the monthly malaria cases and the climate covariates are monthly data. The spatial scale is that of the whole city. Surat is approximately 326 sq. km and Ahmedabad, 471 sq. km. The climate covariates are obtained from a given meteorological station for each city.
Data exclusions	No data excluded.
Reproducibility	We consider two cities with different environmental conditions within the state of Gujarat in NW India. Ahmedabad is semi-arid; Surat is coastal with a maritime influence and is prone to flooding from the Tapi river. Despite these differences the results of the models' comparisons and related analyses are consistent between the two cities. Specifically, the model that includes relative humidity in the transmission rate best explains and predicts the data for each of the cities.
Randomization	NA. The study analyses longitudinal reported cases in a given city.
Blinding	NA. Blinding is not applicable to the time series analyses conducted in this work.

Did the study involve field work? ☐ Yes ☒ No

Reporting for specific materials, systems and methods

We require information from authors about some types of materials, experimental systems and methods used in many studies. Here, indicate whether each material, system or method listed is relevant to your study. If you are not sure if a list item applies to your research, read the appropriate section before selecting a response.

Materials & experimental systems

n/a	Involved in the study
☒	☐ Antibodies
☒	☐ Eukaryotic cell lines
☒	☐ Palaeontology and archaeology
☒	☐ Animals and other organisms
☒	☐ Human research participants
☒	☐ Clinical data
☒	☐ Dual use research of concern

Methods

n/a	Involved in the study
☒	☐ ChIP-seq
☒	☐ Flow cytometry
☒	☐ MRI-based neuroimaging
